# Mini review: linkage between α-Synuclein protein and cognition

**DOI:** 10.1186/s40035-015-0026-0

**Published:** 2015-03-28

**Authors:** Huda Saleh, Ayeh Saleh, Hailan Yao, Jie Cui, Yong Shen, Rena Li

**Affiliations:** Center for Advanced Therapeutic Strategies for Brain Disorders Roskamp Institute, Sarasota, FL 34243 USA; Center for Hormone Advanced Science and Education, Roskamp Institute, Sarasota, FL 34243 USA; Neurodegenerative Disease Research Center, School of Life Sciences, University of Science and Technology of China, Anhui, 230027 China; Beijing Key Laboratory of Mental Disorders, Beijing Anding Hospital, Beijing Institute for Brain Disorders, Capital Medical University, Beijing, 100088 China

**Keywords:** α-Synuclein, Parkinson’s disease, Cognition, Memory, Learning, Alzheimer’s disease

## Abstract

α-synuclein is a protein that plays important roles in cognitive function in the normal brain, although its exact role is not fully understood. However, current studies reveal that defects in α-synuclein function could contribute to various neurodegenerative disorders, such as Parkinson’s disease (PD), a disease with symptomatic progression of deterioration in motor and cognitive function. Recent studies show that the level of α -synuclein in cerebrospinal fluid (CSF) is highly correlated with speed of cognitive decline, suggesting a potential role of α-synuclein in cognitive function. In this mini review, we will be focus on literatures of α-synuclein in cognitive function in the non-diseased brain, as well as the impact that defective α-synuclein has on cognition in disease brain. This will be accomplished by assessing the effects of soluble α-synuclein, α-synuclein oligomers, and extracellular α-synuclein transport, on neurodegeneration.

## Background

α-synuclein aggregates, which interact with various hormones, neurotransmitters, and other macromolecules, are involved in the pathogenesis of many neurodegenerative diseases. There has been an overall consensus that the general cause of many neurodegenerative dementias is due to the improper folding of specific proteins and their accumulation into aggregate species [1-3]. It is well known that α-synuclein plays significant roles in PD. However, it is still unclear when and how these α-synuclein oligomers occur in pathogenesis of PD. Alpha-synuclein localizes to Lewy Bodies, a major pathological marker of PD, and can be detected in various brain regions and nerve nuclei depending on different stages of the disease. For instance, α-synuclein is increased in the dorsal motor nucleus of the vagus nerve, the locus ceruleus, and the substantia nigra during the early phase of PD and in the cerebral cortical areas of advanced PD, ultimately affecting motor skills and causing a decline in cognitive function [1,4,5] Kang and his colleagues, demonstrated that measures of Aβ_1-42_, T-tau (total tau), P-tau181(tau phosphorylated at threonine_181_), and α-synuclein in CSF have prognostic and diagnostic potential in early-stage of PD. Although there are many hypotheses about the role of α-synuclein mechanisms in PD, there is uncertainty on the physiological function of α-synuclein. In PD brain, there is a correlation between protein aggregates in the hippocampus and cognitive deficits, such as volume loss in the cornu armonis (CA2) regions of the hippocampus and related learning impairment. In addition, PD patients with mild cognitive impairments also had defects in the prefrontal cortex and caudate nucleus, suggesting a brain regional atrophy related dysfunction of cognition in PD [6,7]. An interesting study that examined the pathology of cortical and striatal a-synucelin in patients with PD, Parkinson’s disease dementia (PDD), and dementia with Lewy bodies (DLB) and reported that patients with PDD and DLB had greater pathology for cortical and striatal α -synuclein than did patients with PD [8], suggesting a direct association between α -synuclein and dementia.

### α-Synuclein under normal physiological conditions

#### Structure of endogenous α-synuclein in the normal brain

α-Synuclein is a cytosolic neuronal protein from the structurally related synuclein family of proteins. The N-terminal membrane binding domain of synuclein proteins possess a highly conserved motif, which consists of seven 11 residue repeats. Additionally, the synuclein proteins share a natively unfolded tertiary structure. Unlike the N-terminus, the C terminus of synuclein proteins is polar, containing high amounts of charged residues. The α-Synuclein proteins are highly localized at the presynaptic terminals and they have also been expressed in the nucleus and cytoplasmic regions [9]. In its native form, α-Synuclein is present as an unfolded monomer with an approximate size of 14 kDa. When α-Synuclein is bound to lipid vesicles, it acquires α-helical secondary structures, creating a folded tetramer of about 58 kDa [10]. Although extensive molecular studies have been done *in vitro*, the structure of α-Synuclein in cells has not been fully determined.

#### Functions of α-synuclein in normal physiological conditions

Although α-Synuclein has been implicated in numerous cellular processes, the exact physiological function of α-Synuclein remains elusive. However, much research has identified various mechanisms in which α-synuclein interacts with neurotransmitters, lipids, carbohydrates, membrane bound receptors, and other proteins in the brain. Reported binding targets for α-Synuclein includes, phospholipase D2 and protein kinase C. The highly conserved amino-terminal domain of α-Synuclein enables it to interact with synthetic lipid vesicles and it helps to mediate dimerization. In addition, it is known that the polar C-terminus of α-Synuclein undergoes multiple phosphorylation events, indicating a possible regulatory role of the C-terminus. In addition, it has been discovered that α-Synuclein can act as ATP-independent molecular chaperons, which helps to prevent accumulation of denatured proteins [11]. To help determine the normal function of α-Synuclein, an experiment was carried out using α-Synuclein deficient mice. Results from the study supported the theory that α-Synuclein is an activity –dependent negative regulator of dopamine neurotransmission [12]. To further understand the function of α-synuclein expression in normal physiological conditions, a study was conducted using mice with over-expressed α-synuclein. It was found that overexpression of α-Synuclein protected against paraquat-induced neurodegeneration, suggesting that α-synuclein plays a role against toxic insults-induced neurodegeneration [13]. Thus, the predominant normal functions of α-Synuclein in synaptic vesicle trafficking and neurotransmission may be involved.

Current research proposes that α-Synuclein has an indirect role in the soluble *N*-ethylmaleimide-sensitive factor attachment protein receptor (SNARE) proteins, which are involved in vesicle fusion. Under physiological conditions, α-Synuclein initiates the assembly of the SNARE machinery, where it is thought to play a role in the release of neurotransmitters [9]. Furthermore, it has been demonstrated that α-Synuclein inhibits SNARE- mediated vesicle fusion by binding to the lipid bilayer membrane, followed by an indirect interaction between α-Synuclein and the SNARE proteins. The crucial involvement of α-Synuclein in modulating SNARE proteins is supported by an experimental design, which provided insight on the mechanistic action of α-Synuclein in regulating neurotransmission. The study utilized a lipid regulator, arachidonic acid, to stimulate the formation of the SNARE complex and enhance the ability of α-Synuclein to control exocytosis both in *vitro* and in *vivo*. Α-Synuclein was able to sequester arachidonic acid and block the activation of SNAREs [14].

#### Effects of wild type α-synuclein in learning and memory

A popular area of study has been focused on the effects of α-synuclein on cognition. Although α-Synuclein is a mobile protein, it is primarily localized in axons and presynaptic terminals of neurons in the brain regions that are responsible for memory and emotions. A study was done that examined the importance of α-Synuclein in cognition using α-Synuclein knockout mice. Data from this study revealed that the knockout mice had decreased learning abilities in spatial and working memory, thereby showing the significance of α-Synuclein in cognitive processes [15]. Studies investigated the significance of α-synuclein in spatial learning using the hidden platform reference memory version of the water maze in α-synuclein knockout mice. The α-synuclein knockout mice showed no significant short-term learning impairments in learning or probe trails compared with the wild type mice reported from one study [16], while impairments in long term spatial and working memory were observed in α-synuclein knockout mice by another study, suggesting a potential possibility that α-synuclein was necessary for long-term memory specifically [15].

### Role of α-synuclein aggregates in cognition

#### Morphogenesis of α-synuclein during neurodegeneration

α-Synuclein possesses a structural plasticity that is significant to its involvement in neurodegenerative diseases. In addition, defects in the structure of α-Synuclein, such as misfolding, aggregation, and fibrillation factor into the onset of various neurodegenerative diseases. Many of the mutations that are involved in the pathogenesis of PD have been found to occur at the conserved amino-terminal domain of α-Synuclein. When the unstable monomeric α-Synuclein becomes disordered, it can form aggregates that are rich in cross- β-structures. These structures consist of long helical segments that bind to lipid vesicles [17]. Furthermore, the intrinsically unstable α-Synuclein monomer is dynamic, for it can take on a wide variety of conformations, in which molecular crowding can shift the equilibrium in favor of a folded state. There are multiple site of α -synuclein that can be regulated by post-translational modifications (PTMs). The PTMs of α –synuclein alter its conformation, size, and charge, which contributes to the progression of neurodegenerative diseases. There is a wide variety of PTM alterations that occur through numerous processes.

#### Pathogenesis of extracellular α-synuclein

It has been revealed that dopamine (DA) modulates α-synuclein assembly by binding exclusively to the extended conformation of α-synuclein [18]. It was concluded that the loss of dopaminergic transporter in PD brain was a result of neuronal loss [19]. However, it remains unknown whether the loss of DA transported is associated with a-synuclein. Furthermore, another mechanism of α-synuclein is its interaction with lipids, such as cholesterol, which causes a conformational change of a-synuclein. The conformed a-synuclein has a high affinity for cholesterol, which creates a tilted geometry of the cholesterol/α-synuclein complex. In turn, this complex facilitates the formation of an oligomeric channel [3]. The aggregation of α-synuclein oligomers is presumed to be detrimental to cognitive function [20]. Studies show that prolonged exposure to α-synuclein oligomers increases the basal synaptic transmission through the direct binding of α -synuclein to the NMDA receptor activation site. This triggers the firing of calcium-permeable AMPA receptors, which inhibits any response to physiological stimuli. Consequently, this hinders learning and memory, because it impairs long-term potentiation (LTP), which is a function that is carried out in the hippocampus [21]. Recent findings have shown a binding mechanism in which α-synuclein and Aβ peptide directly interact together in a Lewy Body variant of AD (LBV-AD) patients. It was shown that endogenous α-synuclein or recombinant α-synuclein can protect against soluble Aβ_1-42_ induced caspase-3-mediated cortical neurons death via the PI3K/Akt cell survival pathway [22,23]. A binding mechanism has been established in which α-synuclein and Tau bind to DNA in a specific conformation that causes transformational transition and stabilization of Z-DNA conformation [24,25]. Tau and α-synuclein bind to the B-DNA conformation and alter its form by increasing the melting temperature and decreasing the EtBr molecules bound per base pair in the B form of DNA. The altered B form is favored in DNA stability and is a probable intermediate to form the favoring Z-DNA conformation, which are inferred to have a significant role in gene expression in neurodegeneration [26]. Furthermore, it was found that aggregated α-synuclein can directly cross-seed tau fibrillization. Preformed α-synuclein fibrils were assembled from recombinant protein in vitro (primary neurons) and in vivo (transgenic mice). It was concluded that there were at least two distinct strains of synthetic α-synuclein fibrils with differences in the efficiency of cross-seeding tau fibrilization. Proteinase K digestion revealed variance in conformation between these two synthetic strands of α-synuclein, suggesting a potential distinct strain of α-synuclein likely exist in neurodegenerative disease brains [27].

#### Synergistic extracellular and intracellular interactions of α-synuclein on cognition

Past research has been done on solubility of α -synuclein and its effect on cognition. Various immunoreactivities were measured in the frontal cortex of elderly individuals with no cognitive impairment (NCI), mild cognitive impairment (MCI) and early AD subjects. In early AD patients, α-synuclein solubility in the frontal cortex was significantly lower than that in MCI and NCI individuals. There were no differences in soluble α-synuclein levels between MCI and NCI patients. It could be inferred that a decrease in level of soluble α -synuclein may be an early feature of cognitive decline in aging and AD [28]. The levels of mRNA and protein of soluble α -synuclein have been measured in individuals with PDD, DLB, AD and matched controls. It was found that levels of soluble α-synuclein proteins were lower in DLB and PDD subjects. There was no evidence for a corresponding decrease in α-synuclein mRNA levels, which indicates that the alteration of soluble α -synuclein in PD and DLB is at protein level, not at the mRNA level [29]. In addition, a study was done in which extracellular application of α-synuclein oligomers was found to increase intracellular Ca^2+^ levels, induce calcineurin activity, decrease cAMP response and CREB transcriptional activity. This resulted in calcineurin-dependent cell death of the human neuroblastoma cells. Similarly, CaN induction and CREB inhibition were observed when α-synuclein oligomers were applied to organotypic brain slices, which opposed hippocampal long-term potentiation. Furthermore, α-synuclein oligomers induced CaN, inhibited CREB and caused memory impairment in mice that received acute intracerebroventricular injections of α-synuclein oligomers [30]. The general mechanism of secretion of α-synuclein from the intracellular complex to the extracellular complex is still unknown. There are two pathways that are thought to be involved in the secretion of α-synuclein by neurons. One of them involves the non-classical calcium-dependent release of cytotoxic vesicular forms of α-synuclein from transfected SH-SY5Y cells. The other pathway involves the endoplasmatic reticulum/Golgi regulated exocytosis of α-synuclein in primary enteric neurons dependent on sodium channel depolarization of, α-synuclein and brefeldin-A [31]. While the pathology of secreted α-synuclein remains unknown, it appears that α-synuclein can be secreted from neurons into the extracellular space, thereby affecting the homeostasis of neighboring cells. This was demonstrated in a study which found that COS-7 cells treated with exogenously applied α-synuclein secreted from differentiated SH-SY5Y cells, was taken up by dynamin-dependent endocytosis. Upon internalization, α-synuclein increased in the rate of transferrin receptor (TfR) internalization and recycling, a pathology presumed to be involved with PD [32]. Compared to soluble Aβ and tau levels, the soluble α-synuclein levels showed a stronger correlation to cognitive impairment in AD [33]*.* It has been suggested that cognitive dysfunction could be associated with the misfolding of normal soluble α-synuclein by amyloid deposition, suggesting the accumulation of amyloid and α-synuclein caused the loss of solubility in the intracellular cytosolic proteins [34].

### α-Synuclein in neurodegenerative diseases

#### α-Synuclein and AD

Although studies have theorized a potential linkage between α-synuclein and its effects on cognition, the effect of α-synuclein oligomer aggregation on cognition in AD still requires further investigation. One recent study examined the CSF levels of α-synuclein in MCI, AD subjects and healthy controls and found that CSF α-synuclein levels were significantly higher in the MCI and AD groups compared to the control subjects, while the increased α-synuclein levels were associated with decreased Mini-Mental State Exam scores [8]. An interesting study investigated the prevalence of Lewy Bodies (LBs) in AD by using α-synuclein immunohistochemistry. It was found that LBs were found in the amygdala of over 60% early onset AD; thus reducing the functioning of the amygdala in emotional development and motivation, thereby impairing cognitive ability of affected AD subjects [35]. Furthermore, there has been data that revealed an influence of Alzheimer-related lesions on the progression of the neurodegenerative process, in particular, on cognitive decline in both PDD and DLB. This data infers that there is a possible synergistic reaction between α-synuclein, tau, and amyloid beta-peptides, which are the major protein markers of both AD and Lewy Body diseases, and of both vascular pathology and AD [36].

#### Sariations of α-synuclein in AD and PD patients

The discrepancy of the role of α-synuclein in AD and PD patients has been a popular research topic. The quantification of α-synuclein in CSF has been used as a biomarker to distinguish patients with AD, DLB, PD and Multiple System Atrophy (MSA) [37]. For example, in comparison with controls, CSF α-synuclein levels in AD were significantly higher, while PD, DLB and MSA subjects showed significantly lower levels of CSF α-synuclein than AD patients, but they did not significantly differ from CSF α-synuclein levels in the controls [38]. It was also reported that α-synuclein expression was present in the enteric neurons of patients with PD but not in AD patients. An experiment was performed in which α-synuclein was detected in 52% of the general population and its level of expression did not change between ages 40 and 91, while all PD patients with α-synuclein positive and expressed higher α-synuclein levels than that in the age-matched non-PD controls, where AD subjects were no difference from controls [39]. Thus, it can be concluded that either PD develops selectively in the enterically α-synuclein positive subset or PD induces this expression. The absence of increased positive α-synuclein expression in AD subjects indicates that there is a difference in pathogenesis between AD and PD. There has been growing evidence of cell-to-cell transmission of neurodegenerative disease-associated proteins, such as α-synuclein, tau, and amyloid beta, which suggests similarities to the infectious prion protein in spongiform encephaloathies [40]. However, while there is little data on potential human-human transmission of neurodegenerative disease-associated proteins associated with AD, no evidence shows α-synuclein as an infectious protein yet.

### Future studies and current treatment options for α-synuclein aggregation

There are various treatment approaches that prevent α-synuclein aggregation and Lewy Body formation, such as through gene therapy, immunochemistry, and reduction of oxidative stress through diet. One treatment suggests an alternative gene therapy, which requires the transfer of genes that might block α-synuclein accumulation. The β-synuclein, a non-amyloid homologue of α-synuclein, has recently been identified as a potential inhibitor. The in vivo transfer of β -synuclein provides a potential treatment method for PD and DLB [2,41].

Treatment methods have been developed for neurodegenerative diseases such as PD and AD, which are caused by aggregation of specific proteins. This method entails that a patient with such neurodegenerative disorder receives a dose of a high affinity antibody fragment that is immunoreactive with the protein that is aggregated. In turn, this will halt the progression of the neurodegenerative disease of the patient by inhibiting the protein from aggregating [42]. A less conventional treatment method for α-synuclein aggregation involves a dietary treatment option. One study proposed that the neuroprotective mechanism of the major green tea polyphenol, (−)-epigallocatechin-3-gallate can alter brain aging processes and serve as possible neuroprotective agents in progressive neurodegenerative disorders such as PD and AD and assist in the cognitive decline of those who are aging. The properties of green tea polyphenol hinders the reactive oxygen species generation and inflammation of neurodegenrative diseases [43]. There was a similar finding concerning a therapeutic method that reduces oxidative stress and free radical scavenger in neurodegenerative diseases involving an antioxidant mechanism of antioxidant action of lycopene [29].

## Conclusion

The associations of low CSF α-synuclein with poor cognitive function have been used as hallmarks of neurodegenerative diseases, such as DLB, MCI and AD, partially. Studies of animal models with genetic depletion of α-synuclein showed decreased learning abilities in spatial and working memory, suggested a potential possibility that α-synuclein was necessary for non-disease related long-term memory specifically. Together, all suggest that reduced α-synuclein may reflect global impaired neuronal/synaptic function, or non-specific overall cognitive deterioration.
